# Reactive Astrocytes Contribute to Alzheimer’s Disease-Related Neurotoxicity and Synaptotoxicity in a Neuron-Astrocyte Co-culture Assay

**DOI:** 10.3389/fncel.2021.739411

**Published:** 2022-01-21

**Authors:** David Wasilewski, Nelson David Villalba-Moreno, Inke Stange, Markus Glatzel, Diego Sepulveda-Falla, Susanne Krasemann

**Affiliations:** Institute of Neuropathology, University Medical Center Hamburg-Eppendorf, Hamburg, Germany

**Keywords:** primary neurons, reactive astrocytes, co-culture, Alzheimer’s disease, neuroinflammation, synaptotoxicity, neurotoxicity

## Abstract

Pathological hallmarks of Alzheimer’s disease (AD) include deposition and accumulation of amyloid- β (Aβ), neurofibrillary tangle formation, and neuronal loss. Pathogenesis of presymptomatic disease stages remains elusive, although studies suggest that the early structural and functional alterations likely occur at neuronal dendritic spines. Presymptomatic alterations may also affect different CNS cell types. However, specific contributions of these cell types as cause or consequence of pathology are difficult to study *in vivo*. There is a shortage of relatively simple, well-defined, and validated *in vitro* models that allow a straightforward interpretation of results and recapitulate aspects of pathophysiology. For instance, dissecting the AD-related processes (e.g., neurotoxicity vs. synaptotoxicity) may be difficult with the common cell-based systems such as neuronal cell lines or primary neurons. To investigate and characterize the impact of reactive astrocytes on neuronal morphology in the context of AD-related cues, we modified an *in vitro* co-culture assay of primary mouse neurons and primary mouse astrocytes based on the so-called Banker “sandwich” co-culture assay. Here, we provide a simple and modular assay with fully differentiated primary mouse neurons to study the paracrine interactions between the neurons and the astrocytes in the co-culture setting. Readouts were obtained from both cell types in our assay. Astrocyte feeder cells were pre-exposed to neuroinflammatory conditions by means of Aβ42, Aβ40, or *lipopolysaccharide* (LPS). Non-cell autonomous toxic effects of reactive astrocytes on neurons were assessed using the Sholl analysis to evaluate the dendritic complexity, whereas synaptic puncta served as a readout of synaptotoxicity. Here, we show that astrocytes actively contribute to the phenotype of the primary neurons in an AD-specific context, emphasizing the role of different cell types in AD pathology. The cytokine expression pattern was significantly altered in the treated astrocytes. Of note, the impact of reactive astrocytes on neurons was highly dependent on the defined cell ratios. Our co-culture system is modular, of low cost, and allows us to probe aspects of neurodegeneration and neuroinflammation between the two major CNS cell types, neurons, and astrocytes, under well-defined experimental conditions. Our easy-to-follow protocol, including work-flow figures, may also provide a methodological outline to study the interactions of astrocytes and neurons in the context of other diseases in the future.

## Introduction

Alzheimer’s disease (AD) is the most prevalent form of dementia in the elderly and its incidence will significantly increase in the upcoming decades, with an estimated number of 66–76 million by 2030 and 115–136 million by 2050, respectively (Prince et al., 2013; Cummings et al., 2018). AD is a chronic neurodegenerative condition resulting in a progressive cognitive decline, ultimately interfering with daily activities (Weintraub et al., 2012). Pathological features involve deposition of extracellular Aβ aggregates (senile plaques) and build-up of aggregated hyperphosphorylated Tau protein inside the neurons (neurofibrillary tangles), finally leading to a neuronal loss (Cacace et al., 2016). Studies on the genetic AD subtypes affecting the Aβ generation cascade have laid the foundation for the so-called Aβ hypothesis in AD (Cacace et al., 2016; Drummond and Wisniewski, 2017). Soluble and diffusible Aβ species, known as Aβ42 oligomers, play a key role in synaptic loss and synaptic injury in individuals with mild cognitive decline years before the AD diagnosis (Masliah et al., 2001; Scheff et al., 2007; Shankar and Walsh, 2009). The Aβ42 species might cause further synaptotoxicity and neurotoxicity through a vast array of targets and pathways, including a downstream signaling modulating long-term potentiation (LTP), cytoskeletal rearrangement, inflammation, or cell death (Gong et al., 2003; Peineau et al., 2007). Recent observations indicate that neuroinflammation represents a disease element promoting that promotes AD development and progression (De Strooper and Karran, 2016). In pre-clinical AD models, microglia and astrocytes were shown to produce a variety of pro-inflammatory cytokines, suggestive of chronic neuroinflammation, which is apparent even before the development of full-blown disease features (Guillot-Sestier et al., 2015; Heneka et al., 2015; De Strooper and Karran, 2016). Recent failures of clinical trials targeting Aβ hint at limitations of the Aβ hypothesis (Mullard, 2019). Existing shortages of the appropriate pre-clinical models leave the exact disease onset, linearity of disease events, and mechanistic drivers of progression in AD unknown (De Strooper and Karran, 2016; Drummond and Wisniewski, 2017). This, in turn, hampers the translation of research into disease-modifying therapies or biomarkers for preemptive diagnosis (Sperling et al., 2011; Mullard, 2019). *In vivo*, AD mouse models mimic features of AD pathogenesis to a certain degree, such as Aβ plaque deposition, without presenting the whole pathological disease profile (Drummond and Wisniewski, 2017).

*In vitro* models, on the other hand, complement the *in vivo* experiments in several aspects: a) they can recapitulate certain aspects of pathogenesis, such as Aβ-mediated neurotoxicity; b) they reduce biologic complexity, thus, enabling manipulation of cells out of the context of an intricate CNS tissue environment; c) they allow relative control over experimental conditions necessary for compounds testing in a higher throughput manner; and d) they show higher cost and time-effectiveness. In addition to that, *in vitro* models can aid in reducing mouse experiments (3 R principles) (Schlachetzki et al., 2013; Choi et al., 2014).

*In vitro* studies involve the use of a variety of systems ranging from the established cell lines (e.g., HEK293, SH-SY5Y, or PC12 cells) to the primary mouse neurons, *ex vivo* organotypic slice cultures, and, more recently, the use of patient-derived induced pluripotent stem (iPS) cells to obtain CNS cells or “brain organoids” (Schlachetzki et al., 2013; Choi et al., 2014; Amin and PaŞca, 2018). Cell lines exhibit limited neuronal characteristics (i.e., lack of neuronal morphology and differentiations such as well-defined axons, dendrites or synapses, expression of neuronal markers, and presence of a post-mitotic cell state) (Lee et al., 1990; Agholme et al., 2010). Nevertheless, they have been used to study the molecular and cellular processes *in vitro*, since they are easily available and simple in handling. All these methods inherently harbor both advantages and disadvantages (Lee et al., 1990; Agholme et al., 2010; Schlachetzki et al., 2013; Choi et al., 2014; Drummond and Wisniewski, 2017; Amin and PaŞca, 2018). In contrast to cell lines, the primary murine hippocampal or the cortical neurons display hallmarks of neuronal morphology and function (Biederer and Scheiffele, 2007; Beaudoin et al., 2012). Primary neurons display some phenotypic features of neurons, e.g., neurites, extensive connections, and spines, allowing the study of the subcellular protein localization and dynamics, protein trafficking, cell signaling pathways, neurite outgrowth, axon regeneration, synaptogenesis, and synaptic plasticity (Seibenhener and Wooten, 2012; Gomis-Rüth et al., 2014; Schouten et al., 2014). To improve neuronal survival and promote spine maturation, neuron-astrocyte co-culture systems were developed and refined where astrocytes provide tropic support (Kaech and Banker, 2006). One relatively well-described co-culture protocol was published by Kaech and Banker (2006), where postnatal (p0-1) primary hippocampal neurons are grown in defined and serum-free conditions on the underside of a glass coverslip that is suspended over a layer of primary astrocytes.

Several groups have used modifications of this model to study the impact of AD stimulators, and prion disease-related neurodegeneration on the neuronal dendritic spine number and density (Lian et al., 2015; Gottschling et al., 2016). However, it was not investigated if those cues have an impact on astrocytes and thus indirectly contributed to the disease-associated neuronal phenotype. In the present study, we optimized an *in vitro* platform of primary mouse neurons, following a modified bottom-up approach, to model the process of neuronal synaptic degeneration in the context of AD. To study the effects on the neuronal phenotype, as well as to track changes occurring in the astrocytes upon experimental manipulation, primary mouse astrocytes were plated as a feeder layer with a physical barrier between the feeder layer and the neurons. Astrocytes were pre-treated to induce a reactive phenotype. Neurons differentiated for 14 days in co-culture were inverted over the pre-treated astrocytes to measure the indirect effects of the activated astrocytes. Accordingly, we identified the technical key aspects for AD modeling using a neuron-astrocyte co-culture system. We determined the optimal time window for experimental manipulations on both astrocytes and neurons to model the non-cell-autonomous effects on neurotoxicity and synaptotoxicity. Our data show that the astrocytes display an altered morphology and significant changes in their cytokine expression profile that might actively contribute to the disease-associated neuronal phenotype in a co-culture assay in the context of AD-related stimuli. Moreover, we could show that a specific neuron-astrocyte ratio is mandatory to achieve the optimal readout of dendritic synaptotoxicity in the neuron-astrocyte co-cultures.

## Materials and Methods

### Materials

Microscope round cover glasses #1.5 (12 mm Menzel-Gläser 1000 Deckgläser/cover slips) (cat# 11846933, Fisher Scientific, Waltham, MA, United States), staining rack (cat# 8542E40, Thomas Scientific, Swedesboro, NJ, United States); DNase I grade II, from bovine pancreas (cat# 10104159001, ROCHE, Basel, Switzerland); Dumont no. 5 forceps (cat# 11252-23, Fine Science Tools, Heidelberg, Germany); Natriumtetraborat (cat# 11648, RdH Laborchemikalien GbH and Ko KG, Selze, Germany); 2.5% Trypsin (10x), 100 ml, (cat# 15090-046); 0.05% Trypsin-EDTA (1x) (cat# 25300-054, Gibco, Carlsbad, CA, United States); GlutaMAX™-I supplement (cat# 35050-038); B-27 Serum-Free Supplement (50X) liquid (cat# 17504-001 or 17504-044), Neurobasal (cat# 21103-049) were from Gibco or Invitrogen, Carlsbad, CA, United States, and Primocin was from (cat# ant—pm-1, Invivogen, San Diego, CA, United States). The HEPES buffer, 1 M pH 7.3 (cat# 15630-080) and Nunclon Delta Surface, sterile 12 well cell cultures plastic dish (cat# 150628) from Thermo Scientific, Waltham, MA, United States. Tissue culture flask T75 (cat# 83.3911.002, Sarstedt Nürnbrecht, Germany). The 5-Fluoro-2′-Deoxyuridine (FUDR) (cat# F0503-100MG) and Bovine serum albumin (BSA), cat# A9647-50G from Sigma, Saint Louis, MO, United States. The Aβ42, Aβ42-TFA (cat# A-42-T-1), and Aβscr, Aβ-scrambled (cat# A-42-S-1) from GenicBio, Shanghai, China. Paraformaldehyde (PFA) 16% solution, EM Grade (cat# 15710) from Electron Microscopy Sciences, Hatfield, PA, United States. CellTiter 96 Non-radioactive Cell Proliferation Assay (cat# S G4000 and G4100, Promega, Madison, WI, United States). The 4′,6-Diamidino-2-phenylindole (DAPI) Fluoromount-G from (cat#0100-20, SouthernBiotech, Birmingham, AL, United States).

1° Antibodies: Astrocyte marker: anti-GFAP antibody (1:1,000) cat# MAB360, (clone GA5 Millipore, Burlington, MA, United States), anti-GLAST (1:1,000) cat# ab416, Abcam, Cambridge, United Kingdom. Neuronal and synaptic marker: anti-synaptophysin antibody (1:250), cat# ab32594 Abcam; anti-MAP2 antibody (1:500), cat# M9942 and anti-ß-III-Tubulin antibody (1:250), cat#T8328 were both from Sigma, St. Louis, MO, United States; anti-VGlut1 antibody (1:250), cat#135 302, Synaptic Systems, Göttingen, Germany. Amyloidβ antibody: Anti-ß-Amyloid (1:300), 1–16 antibody, cat#9320-02, Previously Covance catalog# SIG-39320, Biolegend, San Diego, CA, United States. Microglia marker: anti-Iba1 antibody (1:1,250), cat# 019-19741, Wako Pure Chemical Industries, Osaka, Japan. Oligodendrocyte marker: anti-Olig2 (1:100), cat# MABN50, Millipore, Burlington, MA, United States; anti-O4, (1:250), Cat# MAB345, Millipore. Fibroblast marker: anti-FSP antibody (1:1,000), cat# ABF32, Millipore; anti-smooth muscle actin antibody (1:1,000), cat# ab5694, Abcam.

2° Antibodies: Goat anti-mouse Alexa 488 (1:500), cat#150113; donkey anti-rabbit Alexa 594 (1:500), cat# 31572; Donkey anti-rabbit Alexa 488 (1:500), cat# A21206; donkey anti-mouse Alexa 594 (1:500), cat#15108 all from Thermo Scientific.

### Animals for Primary Cell Cultures

**FIGURE 1:**
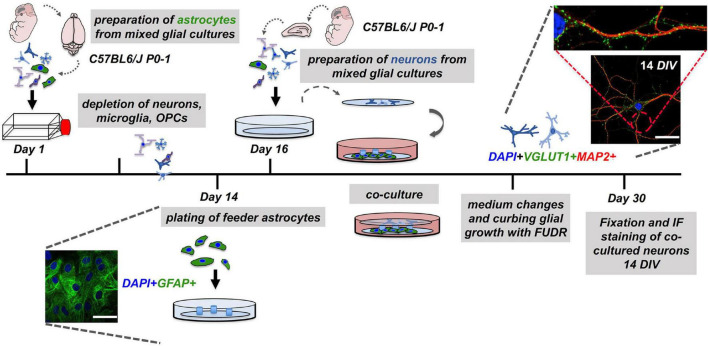
Workflow of the neuron-astrocyte co-culture assay. To set up a neuron-astrocyte co-culture the following steps are necessary: Preparation of mixed CNS cells (dissection #1) for preparation of astrocyte feeder cells cultures must be performed 14 days in advance. The second dissection (dissection #2) of mixed CNS cells served the neuronal cell layer. Here, CNS cells were plated on pre-treated and coated glass coverslips and inverted over the feeder layer. The resulting co-cultures were carefully monitored and treated for the reduction of glia contaminations in the neuronal layer. Co-cultures were subjected to downstream experiments after 14 days in co-culture, which is corresponding to day 30 after the experiment was started. Representative confocal images of neuronal (MAP2), synaptic (VGLUT1), and astrocyte (GFAP) markers are shown (nuclei/DAPI in blue), scale bar = 50 μm.

All research procedures involving animals were according to and approved by the animal care and ethics committee of the City of Hamburg (permit number: ORG739 Molecular Mechanisms of Dementia). Primary mouse astrocyte (dissection #1) and primary mouse neuronal cultures (dissection #2) (Figure 1) were derived from mixed CNS cultures from male or female p0-1 C57BL/6J mice (Charles Rivers, Wilmington, MA, United States), as described by Kaech and Banker (2006).

### Primary Astrocyte Cell Cultures to Obtain Feeder Astrocytes

Proper sterile technique and handling were applied throughout the work with primary cell cultures. Cell culture media or soluble components were filter-sterilized before use. Dissection tools were regularly autoclaved and kept in 70% ethanol (EtOH) after unpacking. Immediately before their use, they were rinsed with dissection medium (DM) containing Hanks’ Balanced Salt Solution (HBSS) 1x, 1% penicillin-streptomycin, 10 mm HEPES, and 0.6% of 45% glucose solution. As in previous protocols, dissection (skin, skull, and brain) was performed with different dissection tools to minimize the contamination risk (Kaech and Banker, 2006; Beaudoin et al., 2012). After decapitation, the head was immersed for a few seconds in 70% EtOH by putting it into a cap of a 50 ml tube, then placed into another plastic falcon filled with chilled DM. Next, the skin flap of the skull was removed using forceps. Tissue preparation was done under a binocular microscope, as shown by Beaudoin et al. (2012). Briefly, separate curved forceps were used for meticulous removal of meninges and of visible blood vessels. It is well-known that insufficient removal of meninges and choroid plexus can lead to contamination with meningeal fibroblasts and endothelial cells (Kaech and Banker, 2006; Beaudoin et al., 2012). For each animal, two halves of the cortex (for astrocyte feeder cultures) and two hippocampi (for neuronal cultures) were obtained and placed in a 15 ml falcon with a chilled DM, then placed on ice. Tissue from four mice was pooled to obtain sufficient cell numbers for the primary cultures. Under the sterile bench, the freshly dissected tissue was transferred with a 5 ml Pasteur pipette into a 15 ml plastic tube on ice. Two milliliters of DM, including tissue pieces, were transferred into a 60 mm dish. Total volume of DM should reach 4.5 ml DM with 0.5 ml pre-warmed 2.5% Trypsin added (Kaech and Banker, 2006; Jones et al., 2012; Schildge et al., 2013). The resulting mixture in 60 mm dishes was covered with parafilm to avoid evaporation and then incubated at 37°C for 15 min in the cell incubator at horizontal agitation of 400 rpm (Schildge et al., 2013). After digestion, 100 μl of sterile 1 mg/ml DNase I was added and was gently swirled. After 1 min, 5 ml of glial growth medium (GGM) (DMEM, 10% FBS, 0.6% v/v from 45% glucose solution, and 1% penicillin-streptomycin) was added to quench the enzymatic reaction (Kaech and Banker, 2006; Jones et al., 2012). Subsequently, gentle trituration with a blue 1,000 μl pipet tip (10 times) was added and the mixture (10 ml total volume) was transferred into a new 15 ml vial. Cells were harvested by centrifugation (5 min at 1,000 rpm). Next, the supernatant was decanted, and 2 ml of GGM was added with a 5 ml plastic pipette to the small cell pellet, which was then gently triturated with a fire-polished glass pipette seven times. After residual tissue pieces were able to sediment, tissue was subjected to another trituration step (seven times). An additional 2 ml of neural maintenance medium (NMM) (Neurobasal medium, 1% 2 mM Glutamax, 2% B27 serum supplement, 100 μg/ml Primocin) was added to the cell suspension. Cells were then centrifuged for 5 min at 1,000 rpm and the resulting supernatant was decanted with the pellet being resolved in 2 ml GGM. A 70 μm cell strainer was placed over a new 50 ml vial and its membrane surface was pre-equilibrated with 500 μl of GGM. Subsequently, the cell suspension was resuspended by gently pipetting up and down 3 times. Cells were then filtered through the cell strainer. Residual cells in the 15 ml tube were washed with an additional volume of 500 ml of GGM and strained into the 50 ml vial. Another fresh 1,000 μl pipette tip was used to aspirate the remaining cell suspension from the bottom side of the cell strainer to harvest additional cells that still adhered to the surface of the cell strainer. Typically, the Hippocampi of the brains of four mice that were dissected and pooled together yield approximately 500,000 cells/ml in 4 ml of a total volume, whereas two cortices yield 1,200,000 cells/ml in 4 ml of total volume. Cell viability was semi-automatically evaluated using a cell counter based on the Trypan blue exclusion and should normally range between 85 and 90%. The cell suspension of the mixed CNS cells was then plated into a T75 cm^2^ tissue culture flask, which was filled up to 12.5 ml with GGM. The next day, the cells were carefully washed with Dulbecco’s Phosphate Buffer Saline (DPBS) 1x, and the GGM medium was changed. Mixed CNS cultures were monitored every second day, with an additional wash step and medium change every 5–7 days.

### Preparation of Astrocyte Feeder Cells

**FIGURE 2:**
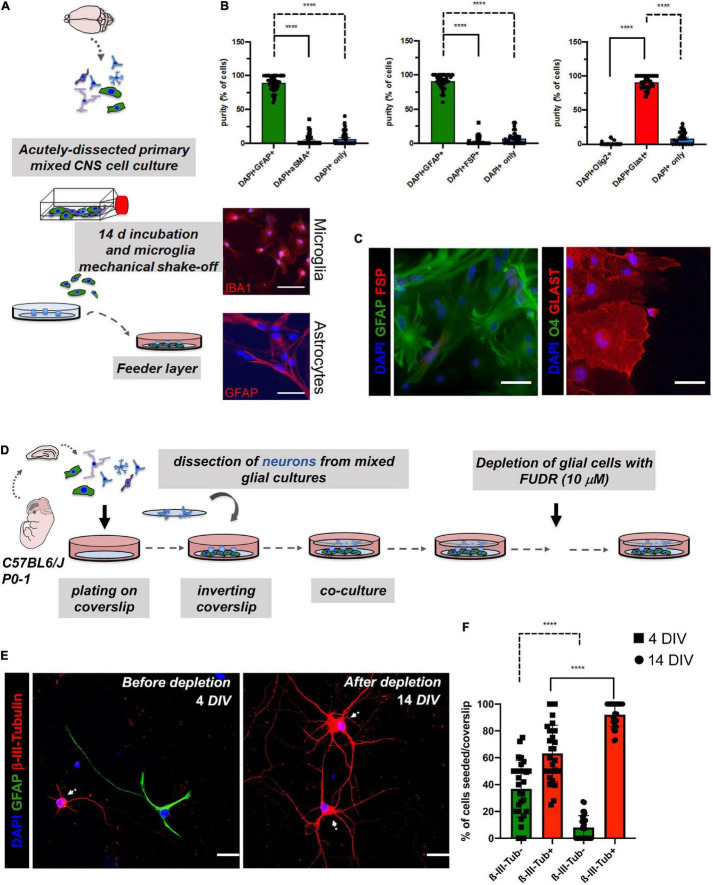
Assessment of potential cell contaminations of the astrocyte feeder layer (>14 *DIV*; lower coverslip) and effect of 5-Fluoro-2′-Deoxyuridine (FUDR) treatment on co-cultures at days 4 of neuron-astrocyte co-cultures (4 *DIV*; upper coverslip) to ensure cell purity of the experimental setting. **(A)** Schematic overview of the generation of the astrocyte feeder layer. Microglia were removed from the astrocyte culture by vigorous shaking. Microglia could then be harvested by centrifugation of the culture supernatant and replated (upper image: representative IBA1 staining of isolated microglia in red; scale bar = 50 μm; DAPI = nuclei in blue). Highly pure astrocytes are still attached to the surface of the culture flask after depletion of the microglia [lower image: representative glial fibrillary acidic protein (GFAP) staining of the purified astrocytes in red; scale bar = 50 μm; DAPI = nuclei in blue]. **(B)** Quantification of double immunofluorescence analyses revealed a purity of the astrocyte layer of ≥ 90%. Here, astrocyte markers (GFAP or GLAST) were co-stained with markers of potentially contaminating cell populations such as fibroblasts (FSP; αSMA) or oligodendrocytes (O4). Data analysis was performed using GraphPad Prism 8 (GraphPad Software Inc., La Jolla, CA, United States), statistical comparison was performed using ordinary one-way ANOVA followed by a *post hoc* Tukey’s test. Data are shown as mean ± SEM. (*N* = 4 mice, with 3 technical replicates each). DAPI staining was used to determine the number of all cells in a given field and to determine the number of cells that were not stained with one of the two markers, respectively. **(C)** For the characterization of the purity of feeder astrocytes, we performed double immunofluorescence analyses. Representative images were taken with a fluorescence microscope. Here, astrocyte markers (GFAP or GLAST) were co-stained with markers of potentially contaminating cell populations such as fibroblasts (FSP) or oligodendrocytes (Olig2); scale bar = 50 μm; *N* = 3 independent experiments. **(D)** Schematic overview of the timeline of FUDR treatment. **(E)** Cells of the upper coverslips (neuronal layer) were examined before FUDR treatment at 4 *DIV* and after FUDR treatment at 14 *DIV* and stained for glia (GFAP) or neuronal markers (β-III-Tubulin) to assess cultures for purity of neuronal cells and contaminating glial cells. White arrows point to neuronal cells. Representative images are shown, scale bar = 50 μm. **(F)** Quantification of the number of non-neuronal cells (β-III- Tubulin-, green columns) vs. neurons (β-III-Tubulin+, red columns) before and after treatment showed a significant increase of neurons. The *N* = 3 independent experiments/condition with technical triplicates, 5 fields imaged per technical replicate, with 30 neurons imaged per condition.

One important modification introduced in this study, for the neuron-astrocyte co-cultures, consists of the incorporation of a second glass coverslip that is placed in the bottom well of a multi-well plastic dish for the lower portion of the co-cultures (astrocyte feeder layer). Glass coverslips of 12 mm in diameter for 24-well plates and 18 mm in diameter for 12-well format were used. Before placing each coverslip into a well of a multi-well dish, dots of heated paraffin were placed on the coverslips in a triangular fashion, which will later provide the physical separation between the lower coverslip (with the astrocyte feeder layer) and the upper coverslips (with the neuronal cell layer) (Figure 1; Kaech and Banker, 2006; Gottschling et al., 2016). The optimal temperature to produce the roundly-shaped paraffin wax dots adhering to the plate was found to be at 110°C. Temperatures below this led to an insufficient adhesion of paraffin wax dots and detachment during the cell culture. At the same time, paraffin should not be too hot to avoid the spreading of the paraffin over the surface of the coverslip (Kaech and Banker, 2006). Subsequently, the prepared multi-well dishes, each is well-equipped with glass coverslips with triangular wax dots, were placed under a sterile bench and irradiated with a UV light for 30 min to minimize the risk of contamination. Before plating the primary astrocytes for co-culture experiments onto the wax dot-coverslips in multi-well dishes, mixed CNS cell cultures were subjected to overnight shaking at 500 rpm at 37°C to reduce the amount of contaminating microglia, as published in previous reports (McCarthy and de Vellis, 1980; Kaech and Banker, 2006; Jones et al., 2012; Schildge et al., 2013). After shaking, the supernatant containing free-floating microglial cells was discarded. Contamination of the astrocyte feeder cells with other cell types was assessed by immunofluorescence analyses and by quantification of the different cell populations (see Figure 2). Of note, the astrocyte cultures with gross contaminations with fibroblasts should be discarded and not used for the co-culture studies. Plating of astrocyte feeder cells originating from the first dissection (dissection #1) was performed regularly for 48 h before the “neuronal” dissection (dissection #2) (see Figure 1). Astrocyte feeder cells were washed 1x with DPBS1x and were detached by trypsin for 10 min. Cells were harvested and the trypsin was removed by spinning down 1,000 rpm for 5 min, with the 80,000 astrocytes being plated per coverslip/well of a 12-well plate on top of an 18 mm wax dot-coverslip (lower coverslip) (see Figure 1).

### Pre-conditioning of Astrocyte Feeder Cells

The medium of the astrocytes was changed 24 h after plating (i.e., 24 h before neuronal dissection), from GGM to NMM (see above), with a total per-well volume of 2 ml, which marks the onset of pre-conditioning of the NMM medium. Astrocytes now start to produce paracrine factors that will subsequently facilitate the growth and maturation of co-cultured neurons (Kaech and Banker, 2006).

### Pre-treatment of Coverslips and Coating of Pre-treated Coverslips for Primary Neuronal Cell Cultures

Importantly, the glass coverslips for neurons (upper coverslip) require a special pre-treatment before use, including the nitric acid-based treatment and banking at 200°C, whereas the coverslips used for astrocytes did not require any treatment (Kaech and Banker, 2006). Nitric acid is used to roughen the surface of glass coverslips which promotes neuron adhesion and differentiation (Kaech and Banker, 2006; Schildge et al., 2013).

Glass coverslips for neuronal cells (18 mm in diameter) were placed into ceramic racks, rinsed with ddH20, and placed in 65% nitric acid for at least 18–24 h (Kaech and Banker, 2006). Subsequently, glass coverslips were rinsed three times for 3 min with ddH2O, placed in 96% EtOH for 30 min, and again rinsed three times for 3 min and 5 min with ddH2O. It was then placed under the sterile bench to dry and to irradiate with UV for at least 30 min. Finally, the treated and dried coverslips were baked at 200°C for at least 4 h. Under sterile conditions, the pre-treated coverslips were then placed into separate wells of a 24-well plate, which was then packed into sterile plastic bags, sealed, and stored under sterile before use. In our experience, the advance production of pre-treated glass coverslips is crucial for the workflow of the assay.

For optimal adhesion, pretreated and baked coverslips were placed in wells of a separate 12-well multi-well dish and further treated with Poly-L-lysine (PLL; 100 μg/ml end concentration) for at least 24 h before cell plating. For this, PLL was dissolved in 1 mg/ml in 0.1 M borate buffer (Kaech and Banker, 2006; Beaudoin et al., 2012). The pH was set to 8.5 (1.9 g borax + 1.24 g boric acid in 400 mL H2O), then filter-sterilized (Beaudoin et al., 2012). Approximately 1 ml of PLL solution (working concentration 100 μg/ml) was added per well and coverslip. Shortly, the PLL was removed with three washes using DPBS1x before plating the neurons. The DPBS1x was left just until the plating (Kaech and Banker, 2006; Seibenhener and Wooten, 2012).

### Dissection of Primary Neuronal Cell Cultures

The cultivation of primary neurons, dissection of brain tissue, and generation of a single cell suspension were carried out as described for primary astrocytes with similar working steps (dissection #2) and with the following differences. Serum-free NMM was used for cultures of neurons. A total of 50,000 or 25,000 freshly dissected CNS cells were plated onto baked and poly-L-lysine-coated coverslips that were placed in wells of a 12-well dish. For plating cells on a 24-well, a format adaption of the number of plated cells was necessary.

### Co-culture Assembly and Propagation of Co-cultures

Co-culturing primary neurons with feeder astrocytes aimed to improve neuronal survival and differentiation up to 14DIV with subsequent experimental manipulation. Briefly, freshly dissected mixed primary CNS cells (dissection #2) were plated onto coverslips that were placed into wells of a 12-well plastic dish. About 1 h after plating, coverslips were inverted and hung over previously prepared feeder astrocytes of another 12-well dish. Importantly, wax dots were placed on the lower coverslip before the astrocyte feeder cell plating. Hence, paraffin wax dots were functioning as spacers to ensure spatial separation between the upper (neurons) and lower (astrocyte) coverslips (Figure 1). Subsequently, 1,000 μl of fresh NMM was added to the remaining volume of the medium. The addition of the mitotic inhibitor 5-Fluoro-2′-Deoxyuridine (FUDR) with a working concentration of 10 μM to deplete other mitotic cell types, such as microglia, oligodendrocytes, and fibroblasts, and to dampen glial proliferation in the upper coverslips, was done only in the very first medium change of the co-culture at day 4 (4 DIV), whereas the changes in the subsequent medium performed every 3 days did not include a mitotic inhibitor (Kaech and Banker, 2006). Co-cultures were monitored daily, and medium changes were performed every 4 days. Importantly, medium changes included 50% removal of an old medium, while the addition of the same volume of fresh medium, using a 1,000 μl pipette to not fully deplete the medium of co-cultured neurons as media, contain paracrine astrocyte-derived factors being critical for proper growth and differentiation of the co-cultures.

### Oligomeric Aβ Preparation, Pre-treatment of Astrocytes With Aβ42, and LPS and Co-culture With Neurons

For induction of reactive astrocytes and assessment of paracrine, non-cell-autonomous neurotoxic, and/or synaptotoxic effects, astrocyte feeders were pre-treated with oligomeric Aβ species; either Aβ42 or Aβ scrambled (Aβscr; negative control). Two millimolar stock solutions of Aβ42 or Aβscr were purchased (GenicBio, Shanghai, China) and prepared according to Falker et al. (2016). Briefly, stock solutions were dissolved in 110 μl ultra-filtered Dimethyl sulfoxide (DMSO) with a subsequent centrifugation step (5,000 × *g* 1 min) to obtain 2 mm stock solutions. Corresponding aliquots were immediately stored in an −80°C deep freezer and thawed directly before the next use. Using this preparation, the starting solution of Aβ mostly contained monomers and small oligomers, but rapidly aggregated into bigger oligomers during the handling (see Falker et al., 2016 for details such as Western Blot or Thioflavin aggregation assay). Of note, other preparations of Aβ, such as mixtures of Aβ40/Aβ42, oligomer preparations, or fibrillary Aβ, might also be used to assess differential effects of diverse Aβ subtypes and aggregation states of astrocytes, as well as their subsequent impact on the neurons. The working concentration for Astrocyte treatment was 2 μM for both Aβscr and Aβ42 (Lau et al., 2014). The LPS was prepared according to Liddelow et al. (2017), and different concentrations were tested with 100 ng/ml as the final LPS concentration for the experiments. After the treatment of the astrocyte feeder layer for 24 h, treatment media was changed to fresh media without LPS or Aβ42 (conditioning). Primary neurons at 14DIV were then inverted over and co-cultured with pre-treated feeder cells for 24 h.

### Immunofluorescence Staining and Microscopy

Immunofluorescence staining was performed to stain fixed cells either for astrocyte and/or neuronal markers (Kaech and Banker, 2006; Beaudoin et al., 2012; Nwabuisi-Heath et al., 2012; Lian et al., 2015). Briefly, after media removal and a washing step with pre-warmed DPBS1x, coverslips were incubated for 10 min with pre-warmed 4% p-formaldehyde (w/v) and 4% sucrose (w/v) fixation solution. After subsequent washing steps, cells were permeabilized by incubating in DPBS1x containing 0.3% Triton X-100 for 10 min, then incubated in a blocking solution (DPBS1x) containing 1% bovine serum albumin (w/v) for 1 h. Primary antibody incubation (see section “Materials” for dilution) was performed for 18 h overnight at 4°C. Coverslips were intensively washed, followed by 2 h incubation with secondary antibodies conjugated to either Alexa Fluor 488 or 594, with subsequent final washing steps in DPBS1x. Changes in astrocyte morphology were visualized by the staining of actin cytoskeleton using the Alexa Fluor™647 Phalloidin (#A22287, Invitrogen by Thermo Fisher Scientific) according to the protocol of the manufacturers after the staining procedure was concluded. After repeated washing steps, mounting was performed with nuclear counterstaining involving 4’,6-Diamidino-2-phenylindole (DAPI) Fluoromount-G. Slides were dried and stored at room temperature in the dark. Images were stored as TIFF files measuring 1,024 × 1,024 pixels with a 63x immersion oil lens objective and a numerical aperture of 1.2 (N.A. = 1.2) using a Leica TCS SP5 confocal laser scanning microscope (Leica Microsystems, Mannheim, Germany). In the case of imaging astrocytes, images were stored as TIFF files measuring 512 × 512 pixels with a 20x objective microscope (Axiovert S100, Carl-Zeiss, Jena, Germany).

### Image Analysis and Readouts

All experiments were independently performed at least three times. For imaging of primary neurons, 15 neurons were imaged per well and coverslip experimental condition (technical duplicates). Three random fields of neuronal dendrites per neuron (zoom = 1.5) were chosen with equal settings maintained in all neurons for each of the three experiments. Astrocytes were imaged as follows: five fields of a given coverslip were imaged (upper, lower, center, right, and left) with three coverslips of the analyzed separate wells (three technical replicates). In total, for experiments with astrocytes, three independent experiments, each with triplicates for each condition, were conducted.

Fluorescence (signal) intensity or integrated density (IntDen) of a given cell marker of interest can reflect protein content and can be used to compare markers in both untreated vs. treated groups of cells. Color threshold and exposure times of a selected marker of interest (e.g., MAP2 or GFAP) were set and maintained constant for each neuron and astrocyte analyzed for each of the three independent experiments. The IntDen values per image were calculated *via* ImageJ according to previous reports (Kaech and Banker, 2006; Marchetto et al., 2010; Gottschling et al., 2016; Odawara et al., 2016). To obtain IntDen values of markers of interest that are specific to neurons or astrocytes, raw confocal and fluorescence images stored as TIFF files had to be converted into and stored as 8-bit images. Images were processed *via* ImageJ (bundled with Java 1.8.0_172) and split into single channels and images. For instance, in the case of neurons, images were split into a red channel (MAP2) and a green channel (GFAP; Synaptophysin—Syn). In the case of astrocytes, the GFAP was used for IntDen quantification and served as a readout for GFAP signal intensity. Sholl analysis served as a readout for the neurotoxicity based on a common cell-fill marker, with MAP2 chosen in this study. As a cell-fill marker, MAP2 depicts the branching pattern of dendrites by plotting the number of branches as a function of the distance from the center of a cell (soma) (Ferreira et al., 2014). Here, we conducted an analysis of linear Sholl plots of our primary neurons. According to Ferreira et al. (2014), we extracted the key indices from linear Sholl plots, including the following: (a) Critical value (Nm), the maximum of the polynomial function, an indicator of maximal branching; (b) Critical radius (rc), the distance at which the Critical value occurs; and (c) Mean value of the polynomial function (Nav), which implies the average number of intersections. We used the Sholl analysis by accessing it as a plug-in from ImageJ14 programmed in Java and bundled in Fiji15. Here, the geometric center (i.e., soma) was manually set and marked using the point tool in Fiji, and the image was analyzed with the Fiji plugins Bitmap Sholl Analysis (Ferreira et al., 2014). Sholl analysis is a technique enabling the quantification of indices of axon length and neurite branching complexity and was carried out with a starting radius of 12 μm progressing with 10 μm intervals to a maximum radius of 120 μm. In addition, the lower threshold was set to 0, while the upper threshold to 128. All neurons of each group were subjected to the same settings.

Synaptic Puncta Analysis (SynPAnal) served for synaptic puncta analysis. Three randomly chosen non-overlapping dendritic segments per neuron were analyzed for quantification of synaptic puncta (Danielson and Lee, 2014). Synaptic puncta were recognized as puncta positive for the presynaptic marker Syn showing specific signal enrichment, and proximity to the dendritic marker MAP2, similarly to previous reports (Kaech and Banker, 2006; Beaudoin et al., 2012; Lian et al., 2015; Gottschling et al., 2016). Spine number and intensity were normalized with an in-built calibration tool provided by the SynPAnal software to the measured length of a given dendritic segment that was tracked with the free-hand selection tool to provide results of the number of synaptic puncta per 100 μm, the density of puncta per μm^2^, and intensity of synaptic puncta per length of 100 μm or per area in μm^2^ (Danielson and Lee, 2014). Soma size was manually examined according to previous studies in the field using confocal 1,024 × 1,024-pixel images (zoom = 1.2x) by outlining the soma in the red MAP2+ channel, omitting dendrites (Daub et al., 2009; Marchetto et al., 2010).

### RNA Isolation and Quantitative Real-Time PCR

Astrocytes were cultured as described above. After treatment with different AD-related stimuli or respective controls for 24 h, the astrocytes were carefully washed in pre-warmed phosphate buffered saline (PBS) and directly lysed in RNA isolation kit lysis buffer. Total RNA was extracted using the mirVana™ miRNA isolation kit (Invitrogen by Thermo Fisher Scientific; Vilnius, Lithuania) according to the protocol of the manufacturer. For the conventional quantitative reverse transcription polymerase chain reaction (qRT-PCR), the total RNA (30 ng) with specific mRNA probes (Applied Biosystems) were used after reverse transcription reaction according to the manufacturer (high-capacity cDNA Reverse Transcription Kit; Applied Biosystems). All mRNA amplifications were performed using the TaqMan Gene Expression Master Mix 5 ml (#4369016) with commercially available FAM-labeled Taqman probes (Applied Biosystems by Thermo Fisher Scientific). Specifically, these are Cxcl10 (Mm00445235_m1), Tnf (Mm00443258_m1), Ccl5 (Mm01302427_m1), Ccl3 (Mm00441259_g1), Cxcl1 (Mm04207460_m1), Ccl2 (Mm00441242_m1), Timp1 (Mm01341361_m1), C3 (Mm00437858_m1), and GAPDH (Mm99999915_g1). The levels of the mRNA were normalized relative to the GAPDH. Real-time PCR reaction was performed using Quantstudio5 (Applied Biosystems by Thermo Fisher Scientific). All qRT-PCRs were performed in duplicates and the data are presented as a relative expression compared to GAPDH as mean ± s.e.m.

### Cytokine Assay

Astrocytes were cultured as described above. After treatment with different AD-related stimuli or respective controls for 24 h, the cell culture supernatant was harvested and cleared from the cellular debris by centrifugation. Cytokine levels in the astrocyte culture supernatants were measured with the Proteome Profiler Mouse Cytokine Array Panel A (ARY006, R&D Systems, Bio-Techne) according to the instructions of the manufacturer. Briefly, the membranes coated in duplicates, with capturing antibodies against 40 cytokines, were blocked and then incubated with the cleared astrocyte culture supernatant in the presence of a cocktail of the biotinylated detection antibodies overnight at 4°C. After washing, membranes were incubated with horse redish peroxidase (HRP)-conjugated antibodies, followed by chemiluminescent detection. The array data, duplicate spots representing the abundance of each cytokine or chemokine, were quantitated using the Licor Image Studio Light software to generate a protein profile, and results are presented as integrated densities.

### MTT Assay

Effects of Aβ42 or LPS treatment on astrocyte survival were probed by performing the CellTiter 96^®^ Non-radioactive Cell Proliferation Assay (Promega, Madison, WI, United States), according to the instructions of the manufacturer (Wang et al., 2012). Briefly, astrocytes were treated for 24 h with different treatment conditions (DMSO 1:1,000, Aβscr 2 μM, Aβ42 2 μM or water, and LPS 10 ng/ml or LPS 100 ng/ml). Experiments were terminated upon the addition of 15 μl of MTT dye solution per well of a 96-well plate, according to the protocol of the manufacturer. Next, after 4 h incubation at 37°C with MTT solution, 100 μl of solubilization/stop solution were added per well. After 1 h, absorbance at 550 and 650 nm were measured using a microplate reader (Molecular Devices, CA, United States).

### Statistical Analysis

Data analysis and visualization were performed with the GraphPad Prism 8 (GraphPad Software Inc., La Jolla, CA, United States). Data were represented as mean ± SD, if not otherwise specified. Ordinary one-way ANOVA for multiple comparisons was used followed by a *post hoc* Tukey’s test. A probability of *p* < 0.05 was considered statistically significant, whereas *p* values higher than 0.05 were considered as non-significant. Non-significant results were not indicated within the bar blots for the sake of readability and clarity of the data. All experiments were repeated at least three independent times (*N* ≥ 3). We performed at least technical duplicates for each experiment and used hippocampal neurons originating from different animals.

## Results

### Co-cultured Neurons Exhibit Signs of Maturity at 14 DIV Offering an Optimal Time Point for Experimental Manipulations

To set-up neuron-astrocyte co-cultures, we first assessed the amount and the viability of our single-celled suspension obtained from a freshly dissected and digested CNS tissue using a semi-automatic cell counter (Countess II Automated Cell Counter, Thermo Scientific, Waltham, MA, United States). The cell isolation protocol is essentially the same for both cell types (astrocytes and neurons). However, astrocytes were derived from the tissue dissection of cortices (cortical astrocytes), while neurons were derived from the tissue dissection of hippocampi (hippocampal neurons). The first dissection served for the generation of a layer of feeder astrocytes, whereas the second dissection is necessary to produce neurons (Figure 1). Minor differences exist with respect to the preparation of both cell types (e.g., nitric acid pre-treatment, baking, and poly-L-lysine coating of glass coverslips for neurons, see section “Materials and Methods”). The mean number of primary cortical CNS cells dissected per brain was 1.135.500 ± 49.797, whereas in the case of hippocampal tissue the number was 279.125 ± 16.543 per mouse per brain (i.e., per 2 hippocampi). The mean life cell fraction in the case of cortical tissue was 90.09 ± 0.8622 %, and in the case of hippocampal tissue, the mean life cell fraction was 93.2 ± 0.5925%. Coverslips with feeder cells were monitored over a time of 2–3 weeks to show the presence of GFAP+ astrocytes that are crucial in providing paracrine (trophic) support to neurons (Kaech and Banker, 2006; Jones et al., 2012; Gottschling et al., 2016). To assess the astrocyte differentiation state of feeder cells, immunofluorescence (IF) staining was performed (Figure 1).

Importantly, feeder cells showed positivity for the pan-astrocyte marker GFAP without the signs of gross contamination with other cells, degeneration, cell death, or overt reactivity during their use for “sandwich” co-cultures. The degree of contamination in the astrocyte feeder layer with other cell types was assessed using the double immunofluorescence staining (Figure 2A). Contaminations with fibroblasts (marker: αSMA and FSP) or oligodendrocytes (marker: Olig2 and O4) were very low or undetectable (Figures 2B,C). Of note, astrocyte cultures with gross contaminations of fibroblasts should be discarded. Since microglia were vigorously shaken off from the astrocyte cells during cultivation, contamination with microglia was also very low or undetectable (Figure 2A). The overall purity of the astrocyte layer in our experiments was ≥ 90%. Over time, we observed a steady growth of feeder cells reaching a sub-confluent to confluent levels typically at 14 days (Figure 1). Neurons pre-treated (nitric acid treated, baked, PLL-treated) coverslip were inverted over the feeder layer of astrocytes that was plated 14 days ago, where wax dots were working as spacers and physical barriers between both cell types (Kaech and Banker, 2006; Jones et al., 2012). Thereby, neurons were directly facing the lower coverslip with the astrocyte feeder layer, but without being in direct contact. Of note, high purity of neuronal cells (on the upper coverslip) was mandatory to assess the consequences of experimental manipulations on the neuronal health and morphology of the neurites and dendritic spines. An overgrowth of the upper layer with glia can impede a reliable analysis of neuronal morphology and downstream neuronal readouts (Kaech and Banker, 2006; Jones et al., 2012). To curb glial overgrowth of the neuronal (upper) coverslip, we implemented a glial depletion step by adding the thymidylate synthase inhibitor and mitotic inhibitor FUDR (working concentration 10 μM) at day 4 (4DIV) (Jones et al., 2012; Figure 2D). Later on, medium changes of NMM did not involve the addition of FUDR in order to avoid adverse effects on neuronal metabolism. Upper coverslips were subjected to IF staining for neuron-specific markers, such as MAP2 or β-III-Tubulin, which enables evaluation of the relative purity of neurons (percentage of β-III-Tubulin+ cells) among the total cell population seeded on top of the upper coverslip (Figures 2E,F). The mean percentage of ß-III-Tubulin+ neurons per field of a given coverslip at 4 *DIV* before glial depletion was 63.2389 ± 21.0176%, whereas, after glial depletion at 14 *DIV*, it was 92.0187 ± 8.8579%. In contrast, GFAP+ DAPI+ astrocytes accounted for 36.7611 ± 21.0176% and 7.9812 ± 8.8579% of all DAPI+ cells at 4 *DIV and* 14 *DIV*, respectively (Figure 2F).

**FIGURE 3:**
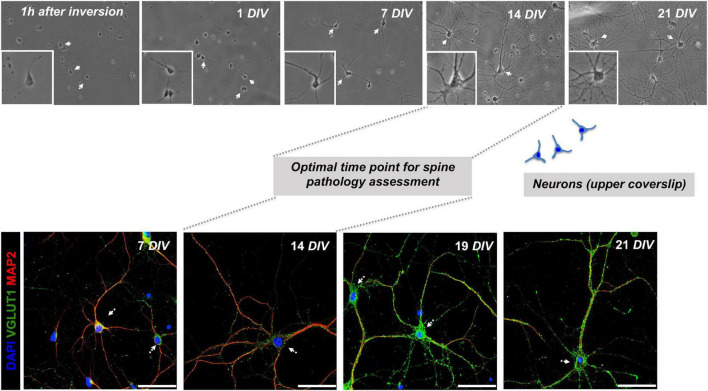
Co-cultured neurons exhibit signs of maturity at *14 DIV*. Monitoring the evolution of primary hippocampal neurons from day 1 up to 21 days in culture (DIV = days *in vitro*) in sandwich co-cultures by serial wide-field analysis (upper row) and fluorescence staining and imaging (lower row). Our study determined the optimal time point for the assessment of dendritic spine morphology of neurons in the upper layer at day 14 of the co-culture. Representative fluorescence images of neuronal (MAP2) and synaptic (VGLUT1) markers are shown, scale bar = 50 μm.

Monitoring by phase-contrast microscopy and IF staining at 7, 14, 19, and 21 *DIV* of upper and lower coverslips, respectively, demonstrated continuous growth and differentiation with increasing age of the co-cultures. As early as 1 h of plating, before the inversion of the upper coverslip over feeder cells, phase-contrast imaging consistently showed the neurons attaching to glass-forming small minor neurites (Figure 3). After 7 days in culture, neurites have grown steadily with some of them resembling axons (Figure 3). Neurons showed signs of maturity *in vitro* at 14 *DIV* with increased soma size, neurite thickness, branching, and an increase in MAP2+ neurites and Syn+ synaptic puncta (Figures 1, 3). Further information, with respect to technical considerations and potential pitfalls of the protocol, are summarized in Supplementary Table 1.

### Neuron-Astrocyte Co-cultures Are Superior to Neuron Monocultures With Respect to Neuronal Growth and Differentiation

**FIGURE 4:**
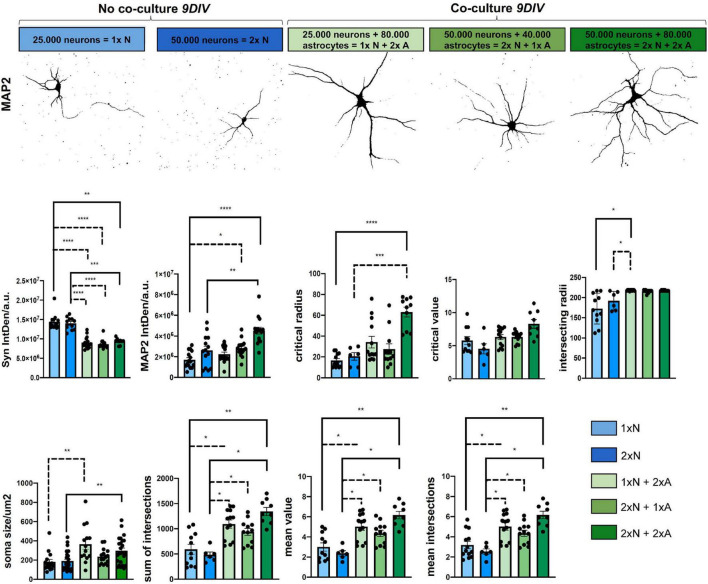
Astrocyte-neuron co-cultures are superior to mono-cultured neurons in terms neuronal differentiation. Analysis of morphological metrics in neurons monocultures, without co-cultured astrocyte feeder cells and neurons co-cultured with astrocytes at 9 *DIV*, show increased differentiation in co-cultured neurons (upper row). Of note, the time point 9 *DIV* was chosen only for the comparison experiments of mono- vs. different co-culture conditions, since neurons in monoculture show a reduced live span *in vitro*. Experiments assessing effects in neuron-astrocyte co-cultures should be performed at 14 *DIV*, when neuronal differentiation in co-culture is optimal. Representative images were taken with a fluorescence microscope (Axiovert 200, Carl Zeiss, Göttingen, Germany). Shown are neurons for the MAP2 channel and displayed in false color for demonstration of the respective morphological hallmarks, scale bar = 50 μm. Determination of IntDen of Synaptophysin signal per field and IntDen of MAP2 signal per field and of soma size are shown. Sholl metrics describe the degree of neuronal differentiation. Shown are critical radius (rc), and critical value (Nm), intersecting radii sum of intersections, mean value, and mean intersections as indicators of complexity and branching of the neurons. For better visibility, only significant differences are shown. Thus, *N* = 3 independent experiments/condition.

To determine basic readouts and to quantify potential advantages of our neuron-astrocyte co-culture setup, we compared neurons in co-cultures vs. neurons in monocultures at 9 *DIV* for morphological parameters. Since neurons in the monoculture were plated in the density that was used throughout our study showed a reduced live span *ex vivo*, and already started to die and disintegrate at around 12 *DIV*, we specifically chose day 9 for the comparison experiment between the mono and co-cultures. Of note, the experiments assessing astrocyte-neuron interaction in the co-cultures should be performed at 14 *DIV*, when neuronal differentiation is optimal (see Figure 3). Readouts were as follows: soma size, IntDen of the fluorescence signal of the neuronal marker MAP2, and the synaptic marker Synaptophysin by immunofluorescence staining and dendritic complexity by Sholl metrics (Figure 4). Moreover, we investigated different seeding densities of neurons in monocultures or co-cultures to optimize our assay (Figure 4). Soma size of neurons was chosen as a readout for neuronal health and differentiation status. Soma size in μm^2^ in neurons in the monocultures, seeded at a density of 25 × 10^3^ neurons/well (1 × N), showed a mean soma size of 180.70 ± 101.53 μm^2^; while neurons plated at the double density (2 × N) showed a soma size of 190.19 ± 97.74 μm^2^, whereas co-cultured neurons showed significantly higher mean values of soma size of 363.90 ± 191.70 for 1x N and 2x A 234.40 ± 80.47 2x N and 1x A and 297.50 ± 138.80 for 2x N and 2x A, respectively (Figure 4). Between the groups of co-cultured neurons [1x N (25 × 10^3^ neurons) co-cultured with either 80 × 10^3^ astrocytes (2x A), 2x N co-cultured with 1x A and 2x N co-cultured with 2x A], there was no significant difference in the mean neuronal soma size (Figure 4). For Sholl analysis, we assessed neuronal dendritic complexity parameters, including mean intersections, mean value, critical radius, and intersecting radii. All these parameters from Sholl analysis are indicators and measures of neuronal health and differentiation and built the basis for further studies. Mean intersections were analyzed *via* the Sholl plugin and showed, again, a significantly increased mean number of intersections of co-cultured neurons as compared to monocultures. The 1x N had 3.185 ± 1.3190 mean intersections, 2x N 2.533 ± 6470, whereas 1x N/1x A co-cultures showed 5.015 ± 1.3440 mean intersections, 2x N/1x A 4.350 ± 1.0492, and 2x N/2x A co-cultures showing with 6.1667 ± 1.1034, the highest number of mean intersections. Likewise, the sum of intersections with 593.1820 ± 328.8418 and 481.6670 ± 138.9239 for 1x N and 2x N monocultures was significantly lower as compared to the values reached in co-cultured neurons. Particularly, 2x N/1x A co-cultures showing 1092.3800 ± 296.4193 sum of intersections and 1x N/1x A 938.167 ± 236.8746 and 2x N/2x A co-cultures showing 1342.22 ± 238.6329 sum of intersections (Figure 4). Key metrics were analyzed as they derive from a polynomial function that can be plotted after running the Sholl analysis plugin, and using a built-in heuristic algorithm in the Sholl analysis plugin, approximating the polynomial function from a linear Sholl plot and, thus, improving local variations of sampled data (das Neves et al., 2021). Interestingly, results of the key metrics were similar to the results above. Significant changes in the mean value were observed between neurons, either subjected to monocultures or co-cultures. For 1x N and 2x N monocultures, it was significantly lower as compared to the values reached in co-cultured neurons: 1x N showed a mean value of 3.0000 ± 1.3505. 2x Na value of 2.3667 ± 0.6345. Conversely, 1x N/2x A showed a Nav of 5.0154 ± 1.3440, 1x N/1x A a mean value of 4.3500 ± 1.0715, and 2x N/2x A showed a mean value of 6.1666 +/-1.1034 (Figure 4). Critical radius and intersecting radii were in line with the results above. In contrast, the so-called critical value showed no significant differences between the mono-cultured or co-cultured neurons (Figure 4). In addition, the signal intensity of the dendritic marker, MAP2, and the synaptic marker protein Syn was assessed by IntDen values. Here, co-cultured neurons generally showed higher MAP2 signal intensity values as compared to neuronal monocultures. However, the mean IntDen values for Syn were higher in monocultured neurons as compared to co-cultured neurons (Figure 4).

### Specific Aβ42-Dependent Reactivity in Primary Astrocyte Feeder Cultures

**FIGURE 5:**
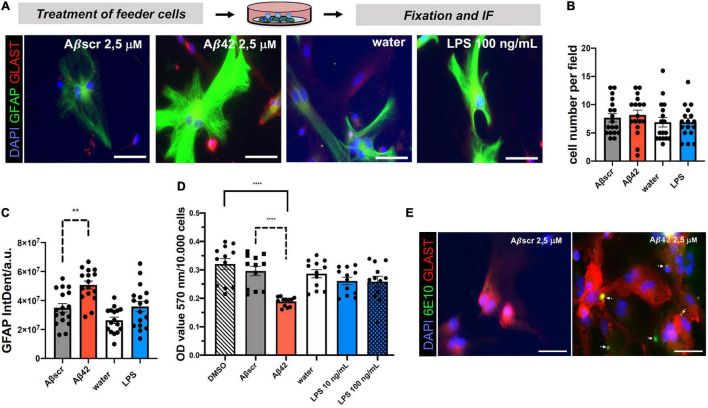
Pre-treatment of primary astrocyte feeder cells with Aβ42 or *lipopolysaccharide* (LPS) induces signs of reactive changes in primary astrocytes. Treatment of monocultures of primary astrocytes with different inflammatory conditions induced a reactive astrocyte characteristic. **(A)** Representative immunofluorescence staining with the astrocyte markers GFAP and GLAST show reactive phenotypes under both treatment conditions Aβ42 or LPS in contrast to the respective controls, scale bar = 50 μm. Evaluation of DAPI+GFAP+ cells per field of coverslips shows no changes in cell number **(B)**, but a significant impact of treatment with Aβ42 on GFAP immunofluorescence signal intensity (IntDen) **(C)** and signal strength by a common MTT assay **(D)**. Absolute OD values for the different treatment and control groups are displayed (D). *N* = 3 independent experiments were performed per condition with two coverslips per experiment per treatment group (i.e., technical replicates). At least 16–18 fields with astrocytes per experimental condition were analyzed. **(E)** Astrocytes in monocultures were treated for 24 h with either Aβ42 or Aβscr at concentrations of 2.5 μM. Astrocytes were stained against astrocytic Glast and with an Aβ42-specific antibody (6E10). White arrows point at Aβ42 positive staining showing binding and/or uptake of Aβ42 by astrocytes in the feeder layer. Images were taken with a fluorescence microscope (Axiovert 200 (Carl Zeiss, Göttingen, Germany), scale bar = 50 μm.

Astrocyte co-culture set-ups have been developed to provide trophic support and increase neuronal health and differentiation *in vitro*. However, paracrine non-cell-autonomous effects and mechanisms of neurotoxicity and synaptotoxicity coming from the astrocyte feeder layer are less well defined. Given the increasing appreciation of the impact of glial cells on neurodegeneration, including the neurotoxicity and synaptotoxicity, we aimed at assessing the effect of toxin-exposed astrocytes on neurons using our assay by pre-treating astrocytes with either Aβ42 or LPS, two known stimulants in the context of AD (Liddelow et al., 2017; Zhang et al., 2017). The water or Aβbscr served as controls, respectively (Figure 5; García-Matas et al., 2010; Li et al., 2016; Pekny et al., 2016; Liddelow et al., 2017). Astrocytes without any neuronal co-culture were exposed to 24 h lasting treatments with either Aβ42 or LPS. Staining with astrocytic markers, GFAP and GLAST, revealed a highly increased intensity for GFAP (Figure 5A). However, the cell number was unchanged by treatment (Figure 5B). Analyses showed that the GFAP IntDen was significantly elevated in Aβ42-treated astrocytes with 5.0685 × 10^7^ ± 110,45,240.84 arbitrary units (a.u.), as compared to Aβscr control with 3.4956 × 10^7^ ± 119,60,517.06 a.u. In contrast, there was a non-significant elevation of GFAP signal intensity upon treatment with LPS with 3.5846 × 10^7^ ± 14517849.33 a.u. as compared to control 2.6356 × 10^7^ ± 8720571.991 a.u. (Figure 5C). In line with that observation, MTT assay of primary astrocytes led to a significant decrease of the optical density (OD) value upon treatment with 2.5 μM Aβ42 compared to Aβscr control, whereas, again, no significant differences in the OD values were detected between water and LPS treatment of astrocytes (Figure 5D).

Microglia, the innate immune cells of the brain, are considered to be the professional phagocytes of the brain. Upon phagocytosis of apoptotic cells from the brain, their phenotype becomes highly dysregulated (Krasemann et al., 2017). Lesser known is that the astrocytes could also significantly contribute to the removal of misfolded proteins or cell debris (Prasad and Rao, 2018). Thus, it was possible that the Aβ-pretreated astrocytes would bind or phagocyte Aβ in our experiments. To assess this, we performed IF staining for Aβ42 (6E10 antibody) and astrocytes (GLAST) (Fu et al., 2015). Astrocytes pre-treated with Aβ42 showed robust signs of positivity for human Aβ42 (Figure 5E). Thus, we speculate that this might also contribute to the astrocyte phenotype.

### Co-culture With Pre-treated Primary Astrocytes Is Associated With Enhanced Synaptotoxicity in Neurons

**FIGURE 6:**
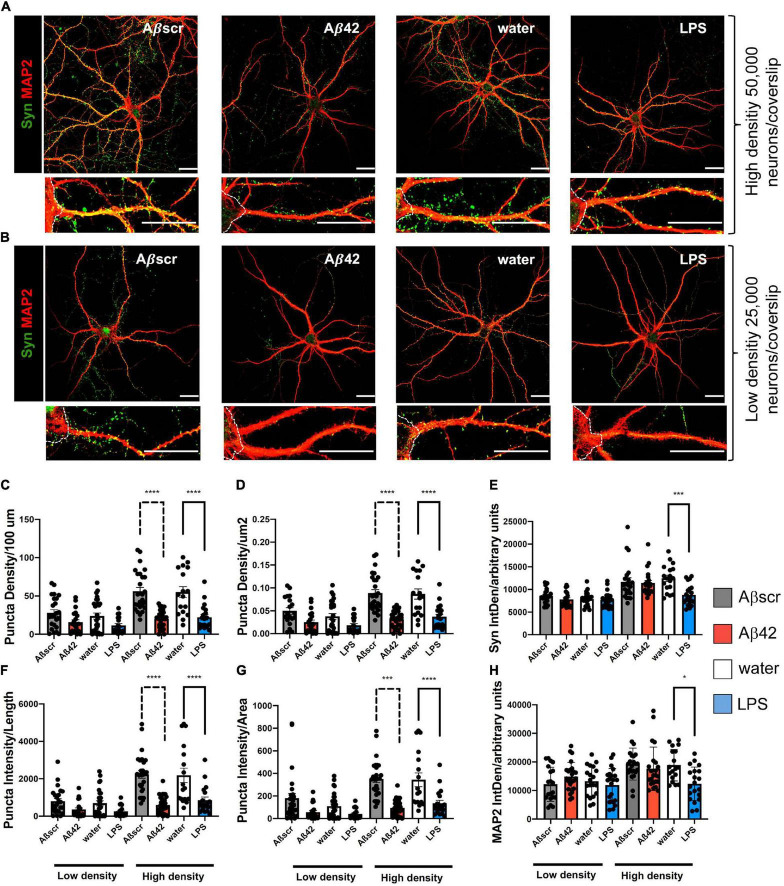
Co-culture of pre-treated primary astrocytes with neurons is associated with synaptotoxic effects. Neurons exposed to reactive astrocytes that were pretreated with LPS (100 ng/ml) or Aβ42 (2.5 μM) show a decrease in synaptophysin Syn+ synaptic puncta as compared to neurons co-cultured with control astrocytes. Water or Aβ42scr served as control, respectively (2.5 μM). **(A,B)** Representative immunofluorescence staining of synaptophysin (Syn) and MAP2 are shown for each condition, scale bar = 25 μm. White dotted lines are marking the neuronal soma. High-density plated neurons **(A)** and low-density plated neurons **(B)** show a decrease in synaptic punctae upon co-culture with Aβ42-pre-treated astrocytes. Puncta density per length (100 μm) **(C)**, puncta density per area (μm^2^) **(D)**, Syn2 signal intensity (IntDen) (A.U.) per field **(E)**, intensity of Syn+ puncta per length **(F)**, intensity of Syn+ puncta per area **(G)** as well as signal intensity of MAP2 (IntDen) **(H)** in arbitrary units (A.U.) per field were analyzed and compared for each of the different treatment groups of neurons. Different treatment groups are sub-divided in both low- and high-density cultures of neurons with respect to initial plating density. Data is shown as mean ± SEM. *N* = 3 independent experiments per condition with at least 18 neurons imaged per condition. Per neuron 3 dendritic segments were randomly chosen. Data included technical duplicates per experiment per condition.

To elucidate whether reactive astrocytes directly impact primary neurons, we placed 14-day-old primary neurons, that were co-cultivated with untreated astrocytes before, over the pre-treated astrocytes for 24 h. To determine the effect of reactive astrocytes ratio on the neuronal phenotype, we used high-density (Figure 6A) and low-density culture neurons (Figure 6B). The Syn+ synaptic puncta (clusters) were used as a readout to analyze the impact of the pre-treatment of astrocytes on neuronal dendritic spines of co-cultured neurons (Figures 6C–H). Analysis of Syn+ synaptic puncta of 3 dendritic segments per neuron, with at least five neurons per experiment, was done in a semi-automatic fashion with SynPAnal with its built-in dendritic area-drawing and thresholding tool. Comparing Syn+ synaptic puncta of co-cultured neurons, either with pre-treated or with naive astrocytes, demonstrated a significant difference in the mean number of synaptic puncta per 100 μm length in neurons after contact with the reactive astrocytes seeded at high density (50 × 10^3^), but not at low density (25 × 10^3^) (Figure 6C). Likewise, the neuron-to-astrocyte ratio (N/A ratio) was significantly associated with the number of synaptic puncta of the co-cultured neurons. Similar results were observed measuring puncta density/μm^2^ based on SynPAnal analysis (Figure 6D). Moreover, the ratio of intensity of Syn+ synaptic puncta and dendritic length or area was assessed. A similar effect on Syn+ synaptic puncta intensity was seen after treatment of neurons with Aβ42 or LPS-pre-treated astrocytes, leading to a significant reduction compared to the control astrocytes (Figures 6F–H).

### Astrocytes Display an Altered Morphology and a Distinctive Cytokine Expression Pattern After Treatment With Aβ42, Aβ40, or LPS

**FIGURE 7:**
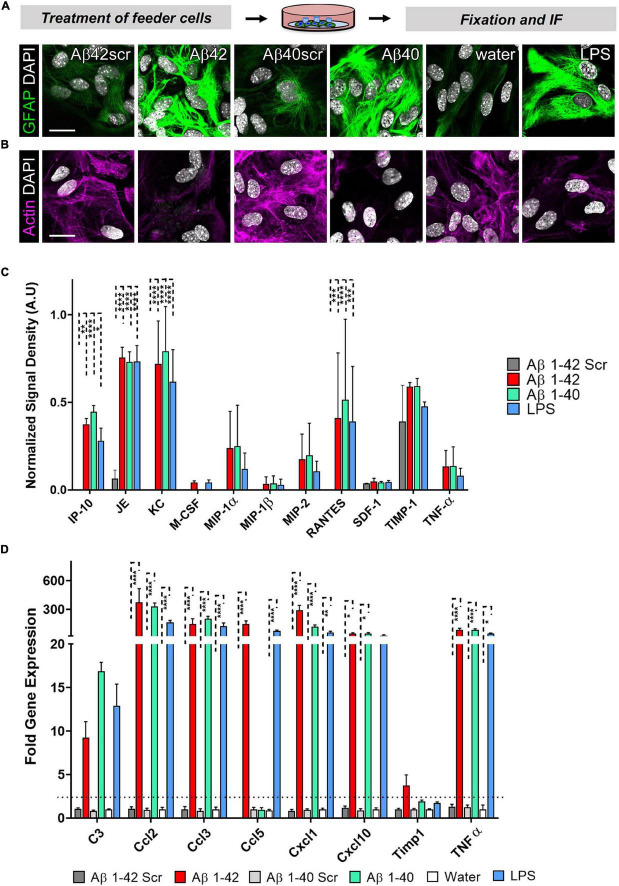
Astrocytes display an altered morphology and a distinctive cytokine expression pattern after treatment with Aβ42, Aβ40, or LPS. Astrocytes were exposed to LPS (100 ng/ml), Aβ40 (2.5 μM), or Aβ42 (2.5 μM), or the respective controls water, Aβ40scr (2.5 μM), or Aβ42scr (2.5 μM) for 24 h. (A+B) Representative immunofluorescence staining with **(A)** the astrocyte marker GFAP or **(B)** AlexaFluor647-Phalloidin for visualization of actin filaments show reactive astrocyte phenotypes with induction of GFAP and cytoskeleton (actin) rearrangement under all three treatment conditions Aβ42, Aβ40, or LPS in contrast to the respective controls, scale bar = 20 μm (*n* = 6; 2 experiments with 2–4 independent replicates each). **(C)** Release of cytokines to the cell culture supernatant after treatment of astrocytes was determined by the Proteome ProfilerTM Mouse Cytokine Array measuring the abundance of 40 different mouse cytokines simultaneously (see Supplementary Figure 1). Positive protein signals were quantified. Significantly upregulated cytokines upon treatment are shown (*n* = 4; 2 independent experiments with 2 replicates each). **(D)** qPCR was performed to measure the expression of selected cytokines including the reactive astrocyte marker complement C3. Relative expression is displayed after normalization against *GAPDH*. Of note, several cytokines are significantly upregulated in treated astrocytes (*n* = 3; 2 experiments with 1–2 independent replicates each).

Since we could show that astrocytes significantly contribute to the neuronal phenotype in an astrocyte-neuron co-culture, we wanted to know more about the activation profile of the reactive astrocytes. Given that Amyloidβ deposits in the brain may comprise different Aβ species. We also included Aβ40 and its respective control, Aβ40scr. Astrocytes were exposed to LPS (100 ng/ml), Aβ40 (2.5 μM), or Aβ42 (2.5 μM), or the respective controls of water, Aβ40scr (2.5 μM), or Aβ42scr (2.5 μM) for 24 h. Astrocytes were then stained for GFAP and actin to display changes in cell morphology. As seen with Aβ42, the Aβ40 treatment and LPS have induced considerable upregulation in the abundance of GFAP (Figure 7A). In contrast, the treatment led to severe changes in the appearance of actin cytoskeleton and reduction of signal intensity (Figure 7B). To assess which factors might contribute to the effect of astrocytes on the neuronal phenotype that we detected (see Figure 6), we performed a Mouse Cytokine Array by measuring the abundance of 40 different mouse cytokines in astrocyte cell culture supernatant of astrocytes that were treated with LPS (100 ng/ml), Aβ40 (2.5 μM), or Aβ42 (2.5 μM), or Aβ42scr (2.5 μM) as control for 24 h. Interestingly, we could detect significant upregulation of several cytokines upon treatment including IP-10/CXCL10, JE/CCL2, KC/CXCL1, and RANTES/CCL5 (Figure 7C and Supplementary Figure 1). Other cytokines like TIMP-1 or TNFα were also elevated but did not reach significance. To confirm our findings, we performed a set of independent experiments and performed qPCR of the pre-selected set of the elevated cytokines from the protein assay in astrocyte cell lysates and included the reactive astrocyte A1 marker complement C3 (Figure 7D). Using this alternative method, we confirmed significant upregulation of IP-10/CXCL10, JE/CCL2, KC/CXCL1, and RANTES/CCL5 in treated astrocytes. Moreover, we could detect significant elevation of MIP1-α and TNFα on the RNA level. Although C3 expression was also increased in treated astrocytes, it did not reach significance.

In summary, we could show that astrocytes secrete a couple of cytokines upon treatment that might directly or indirectly impact neuronal health. We were able to show that astrocytes contribute considerably to the neuronal phenotype in an astrocyte-neuron co-culture. However, this effect is dependent on a specific astrocyte to neuron ratio. Thus, highly controlled experimental conditions are necessary on an *in vitro* co-culture set-up to be able to get reproducible and representative results.

## Discussion

Here, we provided a detailed step-by-step protocol for the set-up of an astrocyte-neuron co-culture with primary cells. We showed that astrocytes not only support neuronal maturation but also directly contribute to the neuronal phenotype. This needs to be put into consideration when studying the effects of chemicals, inhibitors, or stimulators on the neuronal phenotype. Moreover, we could show that this effect is highly dependent on an optimized seeding density of neurons and the astrocyte-to-neuron ratio. To study the effects on the neuronal phenotype, but also track changes occurring in astrocytes upon experimental manipulation, we applied the following modifications to the assay: a) glass coverslips on the dish bottom were equipped with three wax dots arranged in a triangular fashion; b) primary mouse astrocytes were plated as a feeder layer on these wax dot-coverslips serving as a physical barrier between feeder layer and neurons; c) pre-treatment of astrocytes with compounds (here, Aβ42 and LPS) induced a reactive astrocyte phenotype; and d) from an independent experiment, the neurons differentiated for 14 days in a co-culture were inverted over the pre-treated astrocytes for 24 h to model the indirect effects of Aβ42 and LPS on mediated neurons *via* astrocytes. Importantly, this model overcomes some of the difficulties associated with *in vivo* studies as it provides separate access to two main cell types of the CNS, while it retains some complexity allowing experimental manipulations in a dish. Accordingly, we characterized astrocyte-mediated neuronal changes by addressing the following key aspects:

(1) Tracking primary CNS cultures with neurons and astrocyte growth, survival, and differentiation in co-cultures. Thus, determining the optimal time window for experimental manipulations and providing an easy to reproduce step-by-step protocol.
(2) Comparing co-cultured neurons with different ratios of astrocytes vs. neurons in monocultures in terms of neuronal health status and morphometric indices.
(3) Probing the impact of experimental manipulation—including Aβ42 and LPS—on both astrocytes and neurons to model the non-cell-autonomous effects on neurotoxicity and synaptotoxicity.

Regarding our experimental findings, glial cells, such as astrocytes, represent key elements in neurodegenerative and neuroinflammatory conditions. Only recently, Liddelow et al. (2017) showed that astrocytes might attain a reactive or A1 phenotype (Foo et al., 2011; Pekny et al., 2016; Zhang et al., 2016; Guttenplan and Liddelow, 2019). The A1 subtype of astrocytes has been shown to potentially exercise neurotoxic and synaptotoxic properties being induced by microglia-derived pro-inflammatory factors such as IL-1α, TNF, and C1q (Liddelow et al., 2017; Guttenplan and Liddelow, 2019). Of note, we identified several cytokines that are upregulated upon treatment with AD-relevant clues including TNFα. In contrast to the reactive and dysregulated astrocytes, such as A1, recent publications also describe highly beneficial sub-populations of astrocytes, highlighting the diversity of this particular cell type in the brain (Foo et al., 2011; Zhang et al., 2017). For example, Sanmarco et al. (2021) identified an anti-inflammatory subpopulation of astrocytes characterized by the expression of the lysosomal marker protein LAMP12 and the death receptor ligand, TRAIL3, thus, leading to T cell apoptosis under physiological conditions. Advances in the identification of astrocyte functions include their maintenance of brain homeostasis and their reparative and tissue-protective function (Linnerbauer and Rothhammer, 2020). Further studies will help to identify potential stimulators that might be tested in a simplified system, such as our co-culture assay. While our model would be well-suited to test newly identified stimulators or inhibitors, it will probably not be able to determine the heterogeneity within one assay. For these kinds of analyses, more complex culture systems, such as organoids, might be better suited. However, experiments *in vitro* investigated the dysregulated phenotype that is associated with neurodegeneration. Thus, it would be interesting whether astrocytes *in vitro* could be also pushed toward this beneficial phenotype and how this would impact neurons. Our modular modified Banker co-culture assay could help in further characterizing potentially beneficial astrocyte sub-populations by separately treating and studying neurons and astrocytes before combining both cultures to investigate additive effects in the co-culture. Co-culture of neurons with pre-treated astrocytes caused minor changes on Sholl key metrics, whereas synaptic puncta readouts were significantly altered as compared to the control group. This suggests some minor indirect astrocyte-related effects of neuronal dendritic complexity (neurotoxicity) and more pronounced effects related to synaptotoxicity. Interestingly, our co-cultured neurons showed similar numbers of synaptic puncta, like the other groups who reported for neurons in co-culture (Nwabuisi-Heath et al., 2012). Counterintuitively, compared to neurons seeded in higher numbers, in our study, the neurons seeded at a lower density were not able to distinguish and disclose biological effects resulting from different treatments on Syn+ puncta readouts (Figure 4). This might be owing to the fact that neurons *in vitro*, independent of being co-cultured, rely on direct interactions with neighboring neurons. Consequently, in neurons seeded at low densities, biological effects due to treatment might be more difficult to detect since neurons seem to be more vulnerable and already show more variability in growth and differentiation, thus, hampering subsequent treatment readouts. Of note, we showed that a defined astrocyte to neuron ratio is mandatory to study additive effects on neuronal health.

To study synaptotoxicity and neurotoxicity *in vitro*, a common practice is the administration of agents, such as Aβ42 or LPS at similar concentrations, as we used to treat our primary cells. For instance, Lau et al. (2014) used Aβ42 at 5 μM to probe direct synaptotoxic effects of Aβ42 on primary hippocampal rat neurons. Likewise, we used an LPS and Aβ42 to treat primary astrocytes at concentrations that were published earlier by other groups (Lau et al., 2014; Liddelow et al., 2017). To the best of our knowledge, no study probed the indirect effects of LPS and Aβ42 on synaptic puncta readouts by focusing on the impact of astrocytes in a modular experimental setting, as shown by our modified Banker protocol. Since our treatments were aimed at assessing the indirect toxic effect of Aβ42 or LPS, we decided to maintain the relatively low concentrations of known neurotoxic agents, which are known to produce no overt toxicity to astrocytes but could result in more subtle effects such as loss of neurotrophic effects (Lau et al., 2014). The pre-treatment of astrocytes, either with Aβ42 or LPS in serum-free media, was associated with signs of activation, such as upregulation of GFAP and a significant release of cytokines, as compared to control settings (Figures 5, 7). In our study, we used an Aβ42 preparation that we had successfully used in a former study that explored its direct effect on neurons and, thus, with other well-known properties (Falker et al., 2016). However, in future studies, other preparations of Aβ42 and Aβ40, or a combination of both, could also be investigated with regard to their effect on the astrocyte reactivity and their impact on neuronal health, such as oligomeric Aβ-preparation (Walsh et al., 2002).

It is known that CNS cultures involving serum can lead to reactive changes in CNS cells, especially astrocytes, as reported in previous studies comparing prospective astrocyte isolation (e.g., immunopanning or FACS-based isolation) to the classic culture and enrichment of astrocytes according to McCarthy and de Vellis (1980) (Fu et al., 2015; Guttenplan and Liddelow, 2019). In our study, we used the expanded astrocytes using FBS-containing media and later co-cultured them with neurons using serum-free conditions during the time of co-cultures, which likely lowered the impact of previous serum-induced activation of astrocytes and prevented further activation. Nevertheless, with our co-culture protocol, we were able to show consistent dose-dependent responses of astrocytes on Aβ treatment of co-cultures, typically upregulating GFAP when being exposed to Aβ, similar to other pathological conditions, such as stroke and SCI, with signs of hypertrophy (Figure 5; Pekny et al., 2016; Guttenplan and Liddelow, 2019). Future studies with our model for mimicking indirect, astrocyte-mediated neurotoxic, and synaptotoxic effects could include further characterization of the astrocyte feeder layer, including assessment of A1 and A2 marker expression, as shown in previous studies including immunofluorescence labeling or gene expression analysis of C3, Lcn-2 (A1 astrocytes), or S100a10 (A2 astrocytes) (Foo et al., 2011; Fu et al., 2015; Zhang et al., 2016; Krasemann et al., 2017; Liddelow et al., 2017; Prasad and Rao, 2018; Guttenplan and Liddelow, 2019; Sanmarco et al., 2021; Figure 6). Interestingly, the expression of C3 was increased in treated astrocytes but did not reach significance (Figure 7). In contrast, we identified several significantly upregulated cytokines in the treated astrocyte. However, their single or cumulative relevance for neuronal toxicity needs to be determined.

*Bona fide* differentiated primary mouse hippocampal neurons could be obtained with our modified version of the Banker protocol, maintaining neurons up to 3 weeks *in vitro* with co-cultured astrocyte feeder cells providing trophic paracrine support (Figures 1, 3). We implemented a controlled single-dose treatment of the mitotic inhibitor FUDR to co-cultures as done in previous reports to curb the overgrowth of glial cells. In contrast to previous reports or protocols, we quantified the impact of FUDR treatment on the purity of the neuronal (upper) layer of our co-culture to assess the subsequent quality and purity of our neuron preparation (Kaech and Banker, 2006; Jones et al., 2012). We observed that the effect of FUDR in curbing glial proliferation is specific to the upper coverslip, where glial cells (that may have managed to adhere within 1 h after plating together with neurons) may initiate growth due to lack of contact inhibition cues (Kaech and Banker, 2006). Conversely, the lower coverslip, where astrocytes already have reached near confluence before co-cultures are initiated, would almost not be impacted by FUDR treatment. Importantly, we compared FUDR to another commonly used mitotic inhibitor, AraC. In our hands, AraC exerted higher neurotoxicity in our cultures (*data not shown*). This is in line with other reports (Kaech and Banker, 2006; Beaudoin et al., 2012; Jones et al., 2012; Nwabuisi-Heath et al., 2012). Comparison of mono-cultured with co-cultured neurons at 9 *DIV* suggested a significant overall increase in indices that reflect the quality of neuronal cultures and differentiation (soma size, Syn IntDen, MAP2 IntDen) along with dendritic complexity (mean intersections, sum of intersections, mean value, critical radius, and intersecting radii) (Figure 4). Our study provides technical insight and direct comparison of mono-cultured and co-cultured neurons with different ratios of seeded neurons and astrocytes, where an effect-dose relationship of feeder cells in regard to the neurotrophic effects could be observed (Figure 4). Similar to the published literature in the field, our co-cultured neurons show proper signs of differentiation and maturation even before 14 *DIV*, which is seen by many researchers as this time point *in vitro* (Kaech and Banker, 2006; Beaudoin et al., 2012; Jones et al., 2012; Nwabuisi-Heath et al., 2012; Seibenhener and Wooten, 2012; Gomis-Rüth et al., 2014; Schouten et al., 2014; Figures 1, 3).

The aim of our study is to provide a technical backbone to study astrocytes and neurons, both being available for separate manipulations. Here, we used activation of astrocytes with the AD-relevant stimulators, Aβ40, Aβ42, and LPS, for proof-of-principle that it is feasible to perform analyses and significant readouts on both cell types in our setup.

However, such a simplified system comes with the advantages of easy and fast readouts that we described in our study and possible high throughput, but it also has its limitations. The latter concerns the model in general since readouts are very simplified and do not allow further manipulation of other brain cell types such as microglia. Thus, our simplified system will only be suitable to study certain aspects of astrocyte biology. Moreover, the model is useful in rather acute situations as we used it in our study with treatment for 24, but it is not well suited to monitor chronic effects as, but is not well suited to study chronic effects such as the long-term changes that might occur in brain diseases that occur over weeks and months. In future projects, as an alternative and more subtle approach, astrocytes from mice with intrinsic expression of mutated APP isoforms, such as APP-PS1 mice, could be used to investigate their impact on the neuronal phenotype. Another limitation of the murine co-culture system is certainly the fact that we study human disease-relevant cues in a mouse model. Although some pathways of activation might be similar, human brain cells differ considerably from those of mice (Zhang et al., 2016). However, fresh human tissue is very limited. Here, cell culture systems that are developed from human-induced pluripotent stem cells might be the model of choice, but they are very time intensive and relatively costly due to the use of specific differentiation factors.

Future studies with our protocol could involve a bigger panel of neuronal differentiation markers, e.g., using spatial multiplexing technologies such as imaging mass cytometry including panels of different synaptic marker proteins, such as postsynaptic density protein 95 (PSD-95), shank, and vesicular GABA transporter (Vgat), to perform co-localization analyses. Similarly, other downstream assays could be performed, such as cytokine arrays, gene expression analyses, or proteomic analyses. Likewise, the role of extracellular vesicles (EVs) or exosomes as communication devices between astrocytes and neurons or vice versa could be studied in our assay. To date, the exact role of EVs in the CNS is difficult to assess *in vivo* or in organoids due to their complexity and our simplified model-system might thus help to elucidate some of their functions. Specifically, the role of EVs secreted from astrocytes is not well studied yet (Zhao et al., 2021). Current advances in the identification of the EV-secreting cell type may facilitate their specific analysis in the co-culture assay (Vella et al., 2017; Dutta et al., 2021). However, given the small volume of culture media that is used in our assay, it might be difficult to isolate a sufficient amount of purified EVs for analyses. Novel methods to characterize small amounts of EVs, such as imaging flow cytometry for analysis of single vesicles might help here (Ricklefs et al., 2019). Although we studied the role of AD-related stimulators in our project, our model could well be used to study the impact of astrocytes in the context of other neurodegenerative or neuroinflammatory diseases. These include Tauopathies, Parkinson’s disease, Amyotrophic Lateral sclerosis, and multiple sclerosis (das Neves et al., 2021). Here, different isoforms of phosphorylated, aggregated Tau or myelin debris could be used to pre-stimulate astrocytes and investigate their dysregulation and the subsequent effect on neuronal survival. Our model clearly lacks the complexity of mouse models or organoids but provides an easy-to-follow protocol that includes workflow figures and troubleshooting to provide a methodological outline for hands-on work in the laboratory.

### Technical Considerations

Technical considerations and troubleshooting concerning our protocol are summarized in the following table, which can access *via* the Supplementary Table 1.

## Data Availability Statement

The original contributions presented in the study are included in the article/Supplementary Material, further inquiries can be directed to the corresponding author/s.

## Ethics Statement

The animal study was reviewed and approved by Animal Care and Ethics Committee of the City of Hamburg/Germany (permit number ORG739 Molecular Mechanisms of Dementia and ORG1023).

## Author Contributions

DW, NV-M, MG, DS-F, and SK conceived and designed the experiments. DW, NV-M, IS, and SK performed the experiments. SK, DS-F, and MG contributed reagents, materials, and analysis tools. SK and DS-F supervised the study. DW, NV-M, SK, and DS-F analyzed the data. DW and SK wrote the manuscript, with feedback from DS-F and MG. All authors read and approved the final version of the manuscript.

## Conflict of Interest

The authors declare that the research was conducted in the absence of any commercial or financial relationships that could be construed as a potential conflict of interest.

## Publisher’s Note

All claims expressed in this article are solely those of the authors and do not necessarily represent those of their affiliated organizations, or those of the publisher, the editors and the reviewers. Any product that may be evaluated in this article, or claim that may be made by its manufacturer, is not guaranteed or endorsed by the publisher.

## Supplementary Material

Supplementary Figure 1 Cytokines are released by astrocytes after treatment with Aβ42, Aβ40, or LPS. Astrocytes were exposed to LPS (100 ng/ml), Aβ40 (2.5 μM), Aβ42 (2.5 μM), or Aβ42scr (2.5 μM) as control for 24 h. Cleared cell culture supernatant of astrocytes was assessed for abundance of secreted cytokines with the Mouse Cytokine Array. Detection of cytokine expression was performed by chemo-luminescence. **(A)** Representative membranes with dot blots in duplicate for every chemokine/cytokine for each condition are displayed. Note the significant upregulation of several cytokines upon treatment. Quantification of relative upregulation compared to control treatment of selected cytokines is shown in Figure 7. **(B)** Representative identification of blotted chemokines/cytokines in astrocytes treated with Aβ42 using the assay template. **(C)** Reference table of coordinates for cytokine quantification, according to the manufacturer.

Supplementary Table 1 Technical considerations.
