# Supplementary material for: Reactive Astrocytes Contribute to Alzheimer’s Disease-Related Neurotoxicity and Synaptotoxicity in a Neuron-Astrocyte Co-culture Assay
Source: Front Cell Neurosci. 2022 Jan 21;15:739411. doi: 10.3389/fncel.2021.739411 (PMC8813976; doi:10.3389/fncel.2021.739411)
Supplement: Supplementary Figure 1 — Cytokines are released by astrocytes after treatment with Aβ42, Aβ40, or LPS. Astrocytes were exposed to LPS (100 ng/ml), Aβ40 (2.5 μM), Aβ42 (2.5 μM), or Aβ42scr (2.5 μM) as control for 24 h. Cleared cell culture supernatant of astrocytes was assessed for abundance of secreted cytokines with the Mouse Cytokine Array. Detection of cytokine expression was performed by chemo-luminescence. (A) Representative membranes with dot blots in duplicate for every chemokine/cytokine for each condition are displayed. Note the significant upregulation of several cytokines upon treatment. Quantification of relative upregulation compared to control treatment of selected cytokines is shown in Figure 7. (B) Representative identification of blotted chemokines/cytokines in astrocytes treated with Aβ42 using the assay template. (C) Reference table of coordinates for cytokine quantification, according to the manufacturer. [file Image_1.pdf]

Reactive astrocytes contribute to Alzheimer’s disease-related neurotoxicity and synaptotoxicity in a neuron-astrocyte co-culture assay

David Wasilewski, Nelson David Villalba-Moreno, Inke Stange, Markus Glatzel, Diego Sepulveda-Falla, Susanne Krasemann

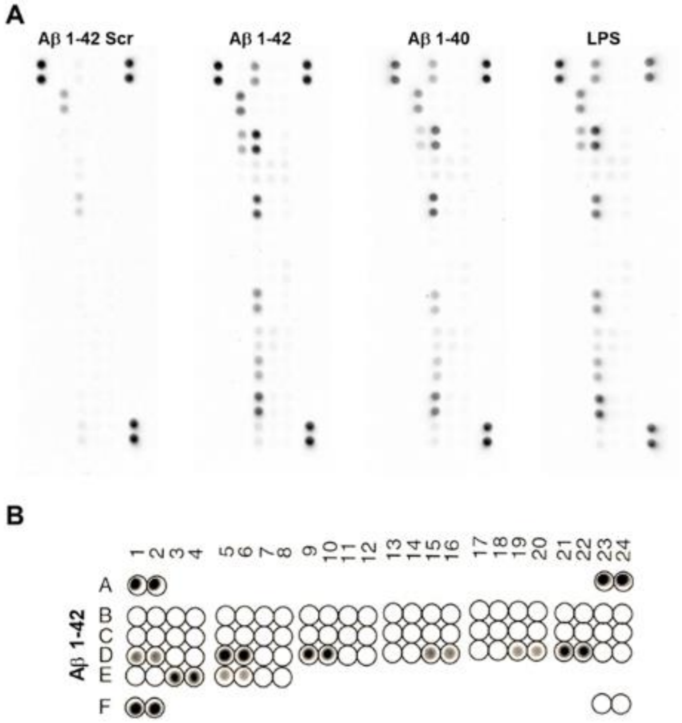

C

Coordinate	Target	Coordinate	Target	Coordinate	Target	Coordinate	Target
A1,A2	Reference Spot	B19,B20	IL-1β	C17,C18	IL-16	D15,D16	MIP-1α
A23,A24	Reference Spot	B21,B22	IL-1ra	C19,C20	IL-17	D17,D18	MIP-1β
B1,B2	BLC	B23,B24	IL-2	C21,C22	IL-23	D19,D20	MIP-2
B3,B4	C5/C5a	C1,C2	IL-3	C23,C24	IL-27	D21,D22	RANTES
B5,B6	G-CSF	C3,C4	IL-4	D1,D2	IP-10	D23,D24	SDF-1
B7,B8	GM-CSF	C5,C6	IL-5	D3,D4	I-TAC	E1,E2	TARC
B9,B10	I-309	C7,C8	IL-6	D5,D6	KC	E3,E4	TIMP-1
B11,B12	Eotaxin	C9,C10	IL-7	D7,D8	M-CSF	E5,E6	TNF-α
B13,B14	sICAM-1	C11,C12	IL-10	D9,D10	JE	E7,E8	TREM-1
B15,B16	IFN-γ	C13,C14	IL-13	D11,D12	MCP-5	F1,F2	Reference Spot
B17,B18	IL-1α	C15,C16	IL-12	D13,D14	MIG	F23,F24	PBS

Supplementary Figure 1

Suppl. Fig. 1 Cytokines are released by astrocytes after treatment with Aβ42, Aβ40 or LPS.

Astrocytes were exposed to LPS (100ng/ml), Aβ40 (2.5µM), or Aβ42 (2.5µM), or Aβ42scr (2.5µM) as control for 24 hours. Cleared cell culture supernatant of astrocytes was assessed for abundance of secreted cytokines with the Mouse Cytokine Array. Detection of cytokine expression was performed by chemo-luminescence. (A) Representative membranes with dot blots in duplicate for every chemokine/cytokine for each condition are displayed. Note the significant up-regulation of several cytokines upon treatment. Quantification of relative up-regulation compared to control treatment of selected cytokines is shown in Figure 7. (B) Representative identification of blotted chemokines/cytokines in astrocytes treated with Aβ42 using the assay template. (C) Reference table of coordinates for cytokine quantification, according to the manufacturer.
